# Supplementary material for: Content-rich biological network constructed by mining PubMed abstracts
Source: BMC Bioinformatics. 2004 Oct 8;5:147. doi: 10.1186/1471-2105-5-147 (PMC528731; doi:10.1186/1471-2105-5-147)
Supplement: Additional File 5 — The original Chilibot query results of the term "long-term potentiation (LTP)" and 22 other terms, limiting the latest references analyzed to the years 1990, 1995, 2000, and 2004. [file 1471-2105-5-147-S5.bz2 › chilibotAdditionalFile5/ltp2004/html/PKA_KV4.2.html]

 


 **PKA** and **KV4.2** 
  
Found 5 abstracts in PubMed,  **5 abstracts were retrieved and analyzed**.  


---

 Search Google  |
 PDF files only 
|  EDU domain only 

---

**Interactive relationship** (e.g. stimulation, inhibition, etc)

- However, this same layer is highlighted by antibodies that recognize  **Kv4.2**  that has been phosphorylated by  **PKA**  at the amino terminus.  Ref: 11040264 Learn MemLearn Mem, 1994
- Therefore, these findings indicate a surprising complexity to kinase regulation of A currents, in that an interaction of two separate molecular events, alpha subunit phosphorylation and the association of an ancillary subunit KChIP3, are necessary for phosphorylation dependent regulation of  **Kv4.2**  encoded A channels by  **PKA** .  Ref: 12451113 J Neurosci, 2002
- For example, the amino terminal  **PKA**  phosphorylation may be acting as a tag for a discrete pool of  **Kv4.2**  to enter stratum lacunosum moleculare.  Ref: 11040264 Learn MemLearn Mem, 2002
- We have developed antibodies recognizing  **Kv4.2**  triply phosphorylated at the three ERK sites as well as two antibodies recognizing singly phosphorylated  **Kv4.2**  channels at the  **PKA**  sites one amino terminal and one carboxy terminal.  Ref: 11040264 Learn MemLearn Mem, 2002
- By using phosphopeptide mapping and amino acid sequencing, we identified  **PKA**  phosphorylation sites on the amino and carboxyl terminal fusion proteins corresponding to Thr 38 and Ser 552, respectively, within the  **Kv4.2**  sequence.  Ref: 10681507 J Biol Chem, 2000
- By using COS 7 cells expressing an EGFP  **Kv4.2**  fusion protein, we observed that stimulation of the endogenous  **PKA**  cascade resulted in an increase in phosphorylation of Thr 38 and Ser 552 within  **Kv4.2**  in the intact cell.  Ref: 10681507 J Biol Chem, 2000

**Parallel relationship** (e.g. studied together, co-existance, homology, etc.)

- These results provide insight into likely sites of regulation of  **Kv4.2**  by  **PKA** .  Ref: 10681507 J Biol Chem, 2000
- Taken together, these results demonstrate that dendritic, A type K channels are dually regulated by  **PKA**  and PKC through a common downstream pathway involving MAPK, and the modulation of these K channels may be accounted for by the phosphorylation of  **Kv4.2**  subunits.  Ref: 12077183 J Neurosci, 2002
- We have previously shown the existence of two  **PKA**  phosphorylation sites in the  **Kv4.2**  sequence.  Ref: 12451113 J Neurosci, 2002
- In the present studies we made the surprising finding that  **PKA**  phosphorylation of the  **Kv4.2**  alpha subunit is necessary but not sufficient for channel modulation.  Ref: 12451113 J Neurosci, 2002
- We determined if  **Kv4.2**  alpha subunits were directly phosphorylated by cAMP dependent protein kinase  **PKA** .  Ref: 10681507 J Biol Chem, 2000
- Phosphorylation consensus sites for various kinases exist within the sequence of the potassium channel subunit  **Kv4.2** , including consensus sites for extracellular signal regulated kinase mitogen activated protein kinase ERK MAPK, protein kinase A  **PKA** , protein kinase C PKC, and calcium calmodulin dependent kinase II CaMKII, and kinase assays have shown that particular amino acids of the consensus sites are bonafide phosphorylation sites in vitro.  Ref: 11040264 Learn MemLearn Mem, 2000
- We also observed modulation of  **PKA**  phosphorylation at these sites within  **Kv4.2**  in hippocampal area CA1.  Ref: 10681507 J Biol Chem, 2000
- For example, the stratum lacunosum moleculare, which receives inputs from the entorhinal cortex via the perforant pathway, displays relatively little ERK phosphorylated  **Kv4.2**  or  **PKA**  carboxy terminal phosphorylated  **Kv4.2** .  Ref: 11040264 Learn MemLearn Mem, 2000
- **PKA**  modulation of  **Kv4.2**  encoded A type potassium channels requires formation of a supramolecular complex.  Ref: 12451113 J Neurosci, 2002
- Similarly, of the three antibodies tested, the soma of CA3 neurons are primarily recognized by the ERK triply phosphorylated  **Kv4.2**  antibody, and the mossy fiber inputs to CA3 are primarily recognized by the carboxy terminal  **PKA**  phosphorylated  **Kv4.2** .  Ref: 11040264 Learn MemLearn Mem, 2002
